# Miliary tuberculosis with bilateral adrenal involvementand adrenal insufficiency

**DOI:** 10.1590/0037-8682-0004-2026

**Published:** 2026-04-10

**Authors:** Mustafa Yeşilyurt, Adem Karaman

**Affiliations:** 1 Atatürk University, Faculty of Medicine, Department of Radiology, Erzurum, Turkey.

**FIGURE 1:**
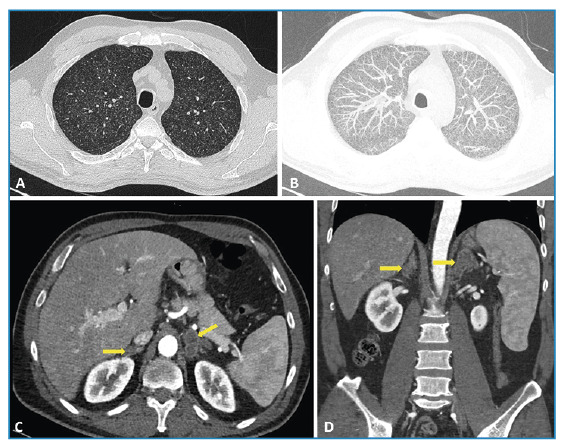
**(A)** Axial high-resolution computed tomography image showing numerous randomly distributed pulmonary micronodules. **(B)** Maximum intensity projection image demonstrating a diffuse miliary pattern throughout both lungs, consistent with miliary tuberculosis. **(C)** Axial contrast-enhanced abdominal computed tomography showing bilateral adrenal gland enlargement with hypodense, mass-like thickening (arrows). **(D)** Coronal reformatted image confirming symmetric bilateral adrenal involvement (arrows).

A 51-year-old man presented with persistent cough, fatigue, and intermittent abdominal discomfort. Microbiological examination of sputum using an automated mycobacterial growth detection system demonstrated growth of *Mycobacterium tuberculosis*, and the interferon-gamma release assay (QuantiFERON-TB Gold) was positive. High-resolution computed tomography of the thorax showed numerous diffusely distributed micronodules consistent with a miliary pattern of pulmonary tuberculosis (Figure 1), reflecting hematogenous dissemination. To evaluate possible extrapulmonary involvement, contrast-enhanced abdominal computed tomography was performed and demonstrated bilateral adrenal gland enlargement with hypodense, mass-like thickening (Figure 1). The lesions were relatively symmetric and lacked imaging features suggestive of metastasis or acute hemorrhage. In the clinical context, these findings were considered consistent with adrenal tuberculosis. Hormonal evaluation revealed elevated plasma renin concentration (177 µIU/mL) and adrenocorticotropic hormone levels (107 pg/mL), with relatively low aldosterone levels (70 pg/mL), supporting primary adrenal insufficiency.

Adrenal tuberculosis is an uncommon manifestation of extrapulmonary tuberculosis and is most often associated with disseminated or miliary disease^1^. Bilateral adrenal involvement is typical and may lead to primary adrenal insufficiency due to progressive glandular destruction^2^. This case underscores the importance of evaluating adrenal glands in patients with miliary tuberculosis, as combined imaging and hormonal assessment may enable early recognition of adrenal insufficiency and help prevent life-threatening complications.
